# A Novel Thrombolytic Regimen for Mechanical Prosthetic Valve Thrombosis in a Patient With Antiphospholipid Syndrome

**DOI:** 10.7759/cureus.23979

**Published:** 2022-04-09

**Authors:** Philippe F Nyembo, Kevin G Buda, Abel Hooker, Woubeshet Ayenew

**Affiliations:** 1 Internal Medicine, Hennepin Healthcare, Minneapolis, USA; 2 Cardiology, Hennepin Healthcare, Minneapolis, USA

**Keywords:** systemic thrombolysis, tissue plasminogen activator (tpa), resistant thrombus, hypercoagulable state, mechanical prosthetic valve thrombosis

## Abstract

Management of mechanical prosthetic valve thrombosis (PVT) includes medical and surgical options. Standard medical treatment involves thrombolytic therapy with repeated slow infusions of low-dose IV tissue plasminogen activator (t-PA). The evidence for managing mechanical PVT that does not respond to the standard t-PA dosing is limited in the setting of an exacerbating hypercoagulable condition. We present a case of a patient with a history of antiphospholipid syndrome who presented with a probable thromboembolic myocardial infarction secondary to a mechanical mitral valve thrombosis that did not improve with systemic anticoagulation and repeated standard t-PA dosing but rapidly resolved with ultraslow, high-dose t-PA.

## Introduction

Thrombus formation is an infrequent but well-recognized complication in mechanical prosthetic heart valves [1]. Symptomatic mechanical prosthetic valve thrombosis (PVT) has high morbidity and mortality [2]. PVT management often involves fibrinolysis with slow infusions of low-dose tissue plasminogen activator (t-PA), which clears thrombus in over 90% of patients [3]. However, hypercoagulable states can lead to resistance to the standard fibrinolytic therapy [4,5]. To our knowledge, our report is the first to describe successful thrombolytic therapy with ultraslow, high-dose t-PA for mechanical PVT resistant to standard dosing in a patient with triple-positive antiphospholipid syndrome (APS).

## Case presentation

History of presentation

A 28-year-old female presented to Hennepin Healthcare with three days of intermittent dull chest pain. The chest pain was associated with mild shortness of breath on exertion. She denied orthopnea and paroxysmal nocturnal dyspnea. She reported normal functional capacity without previous angina or shortness of breath since undergoing cardiac surgery for valve replacement one month before developing the symptoms. 

Medical history

The patient had a history of triple-positive antiphospholipid syndrome, distant ischemic stroke, and a St. Jude mechanical valve (St. Jude Medical Inc., Minneapolis, Minnesota) replacement one month before presentation for severe mitral valve regurgitation from nonbacterial vegetation and valve thickening. The patient was on warfarin for antithrombotic therapy and was adherent.

Differential diagnosis

The most likely differential diagnosis on arrival included pulmonary embolism, acute coronary syndrome, and pericarditis.

Investigations 

**Figure 1:**
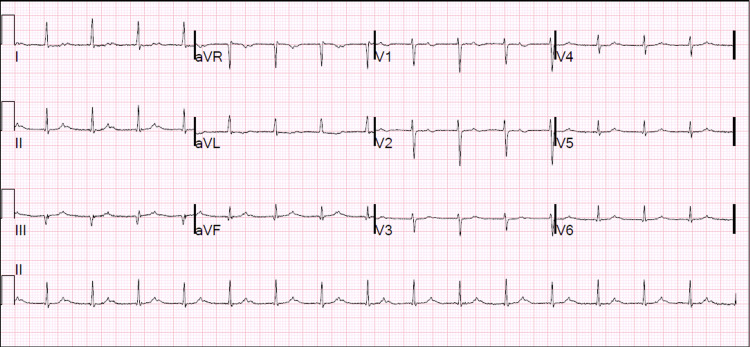
Electrocardiogram on admission Initial electrocardiogram showing sinus rhythm with first degree AV block; nonspecific changes in inferolateral T waves suggesting possible ischemia

**Figure 2:**
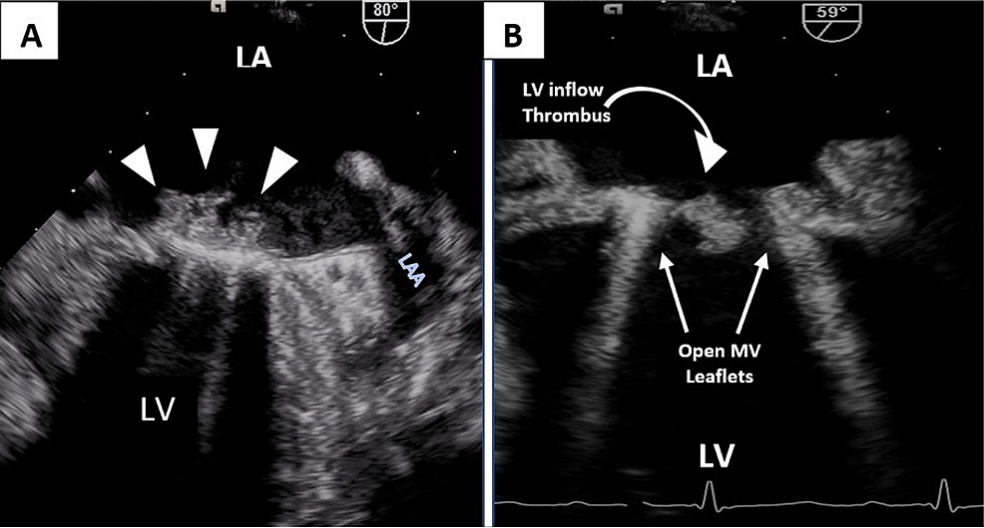
Transesophageal echocardiogram Initial transesophageal echocardiogram showing (A) multiple sessile thrombi (arrowheads) on mitral valve annulus and (B) diastolic protrusion of a large mobile thrombus (curved arrow) into LV inflow between open prosthetic MV leaflets. LA = left atrium, LAA = LA appendage, LV = Left ventricle, MV = mitral valve

On arrival, the patient was afebrile with normal pulse and blood pressure. Oxygen saturation was 98% on room air. Examination revealed a prosthetic closing click with S1 and regular S2 without a murmur. ECG showed diffuse nonspecific T wave abnormalities (Figure 1). Troponin I increased from 2.8 mcg/L on presentation to a maximum of 3.3 mcg/L (ref: ≤ 0.030 mcg/L). A transthoracic echocardiogram (TTE) revealed mildly reduced left ventricular systolic function with a new mid to basal inferior wall hypokinesis and elevated trans-prosthetic mean gradient but poor visualization of the valve due to prosthetic artifacts. Although the INR was supratherapeutic at 4.2 on admission, the chromogenic factor X (CFX), which is used to monitor oral anticoagulation in patients with conditions that cofound INR levels, was elevated at 43% (therapeutic range: 25%-30%) despite confirmed compliance with warfarin therapy. The CFX level of 43% correlates with an INR of 2 or below which for this patient was subtherapeutic [6]. These findings raised concerns for thromboembolic myocardial infarction from mechanical PVT. A transesophageal echocardiogram (TEE) confirmed a large thrombus burden along the prosthetic annulus and the mitral leaflets with diastolic protrusion of a thrombus into the left ventricular inflow. The annular mass's low echodensity and irregular shape were atypical for pannus. Normal excursion of both leaflets was seen (Figures 2A-2B). The trans-prosthetic mean gradient was elevated at 13.7 mmHg with a heart rate of 95 beats per minute. 

Management

**Figure 3:**
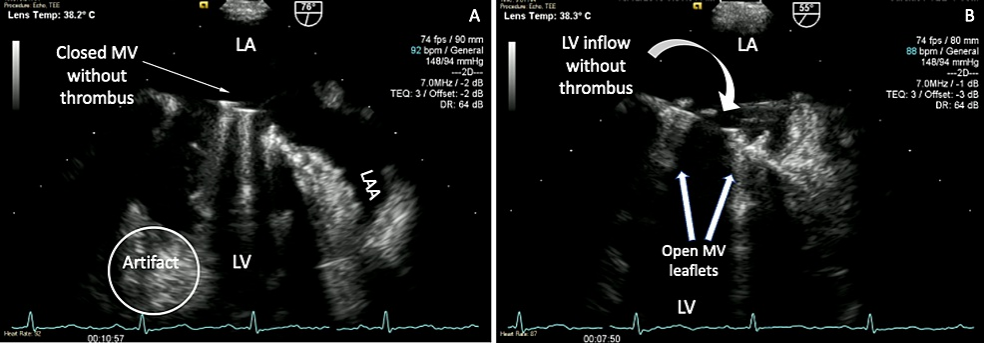
Transesophageal echocardiogram demonstrating thrombi resolution after thrombolysis with ultraslow high-dose t-PA Transesophageal echocardiogram showing (A) closed prosthetic mechanical mitral valve with resolution of the previously seen sessile thrombi (one arrow). (B) LV inflow with prosthetic mechanical mitral valve strands (curved arrow) and open prosthetic mitral valve leaflets (two arrows) without the previously seen large mobile thrombus. LA = left atrium, LAA = left atrial appendage, LV = left ventricle, MV = mitral valve

**Figure 4:**
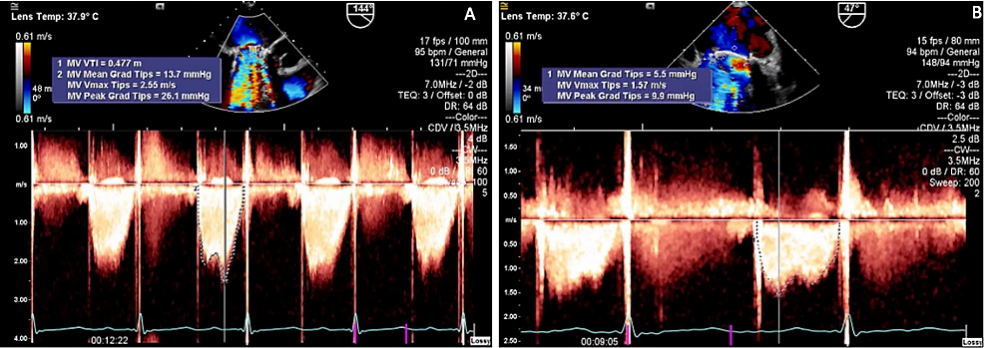
Transesophageal Doppler echocardiogram demonstrating normalization of trans-prosthetic mean gradient after thrombolysis with ultraslow high-dose t-PA Transesophageal Doppler echocardiogram showing on the left (A) a trans-prosthetic mitral mean gradient of 13.7 mmHg at a heart rate of 95 beats per minute prior to thrombolysis with the ultraslow high-dose t-PA; on the right (B) notice normalization of the trans-prosthetic mitral mean gradient (5.5 mmHg) at a heart rate of 94 beats per minute after thrombolysis with the ultraslow high-dose t-PA. t-PA=tissue plasminogen activator, MV=mitral valve

**Figure 5:**
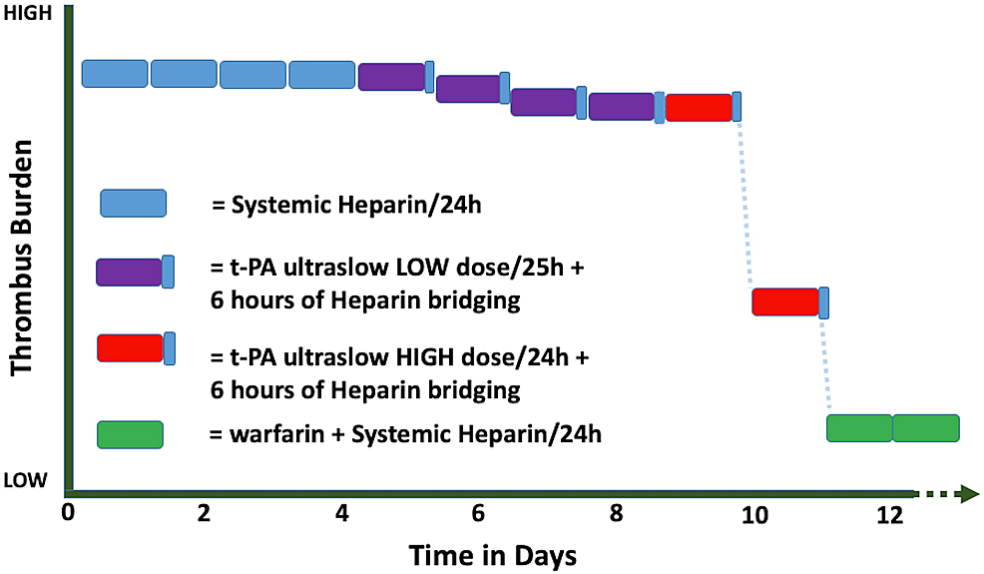
Graphic representation of Thrombus burden response to various anticoagulation and fibrinolytic regimens

Therapeutic heparin infusions over four days and fibrinolysis with four cycles of standard ultraslow low-dose t-PA (25mg infused over 25 hours) did not resolve the thrombus burden. Following two cycles of ultraslow high-dose of t-PA (100mg infused over 24 hours), there was complete resolution in thrombus burden and normalization of trans-prosthetic mean gradient (Figure 3A-3B; Figures 4A-4B; Figure 5).

Follow up

**Figure 6:**
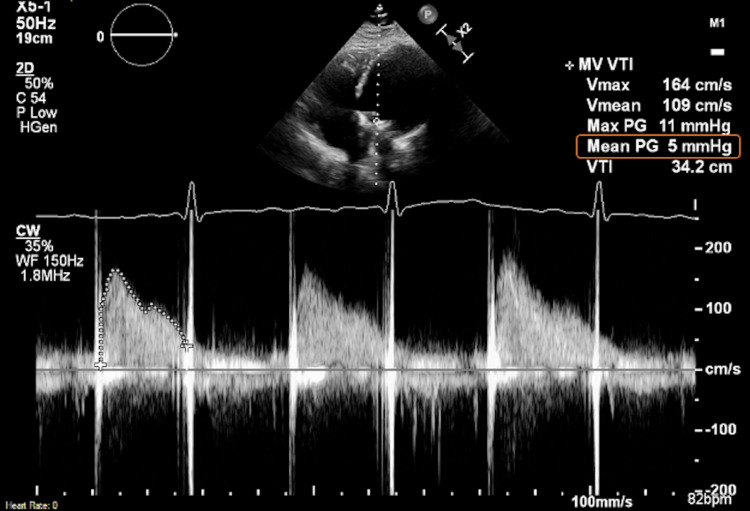
Transthoracic Doppler echocardiogram at six months follow up Transthoracic Doppler echocardiogram at the six months follow up showing a trans-prosthetic mitral valve mean gradient of 5 mmHg at a heart rate of 82 beats per minute.

The patient had no bleeding complications or evidence of recurrent thromboembolism. She was discharged home on warfarin and ASA and had an unremarkable post-hospital course over six months of follow-up (Figure 6). 

## Discussion

The risk of PVT increases significantly in the mitral position and in the setting of hypercoagulable states such as APS [1,4]. Mitral valve PVT is five times more common than aortic valve PVT, with an incidence of up to 0.5 per 100 patient-years and 0.1 per 100 patient-years respectively [7]. APS is associated with the presence of antiphospholipid antibodies that increase the risk of venous and arterial thrombosis through several mechanisms. Activation of endothelial cells and platelets, as well as inhibition of naturally occurring antithrombotic proteins like protein C, are cited mechanisms of thrombosis secondary to APS [5,8]. Additionally, the presence of specific antiphospholipid antibodies (lupus anticoagulants) in APS can erroneously elevate the INR levels by binding to phospholipids in laboratory in-vitro tests [9]. On the contrary, CFX is a test that measures the activity of factor X (a vitamin-K-dependent coagulation factor) independently of phospholipids. Thus, CFX is the preferred test to monitor warfarin therapy in patients with APS [10]. Thrombolytic therapy (TT) with slow-infusion, low dose fibrinolytic, or emergency repeat valve surgery are currently the recommended first-line treatments in patients with symptomatic left-sided obstructive mechanical PVT [11]. Over the past decade, TT was shown to be a reasonable alternative to repeat valve replacement for PVT, with success rates surpassing 80% [12]. There is no universal definition of failed TT. In one study, TT was considered failed if the thrombus had not resolved after two to three successive doses of a fibrinolytic agent [13]. Although surgical management is an option for patients who fail TT, urgent surgery of PVT carries a significant risk of mortality (» 20% for surgery compared to » 2.8% for slow, low dose t-PA regimens) [13,14]. The safe use of higher doses of t-PA after failed initial TT with repeated standard low doses has not been widely described [15]. 

Our patient presented with a probable embolic myocardial infarction and features of high thrombus burden despite excellent adherence to anticoagulation and an INR in the target range. The risk of prosthetic mitral valve thrombosis was compounded by the presence of APS. A high prevalence of hypercoagulable conditions has been described in patients with PVT [4]. However, no studies describe TT for PVT in this patient population, including those with APS, where the response to fibrinolysis may be impaired [5]. Perhaps, this can explain the lack of response in thrombus burden despite standard repeated ultraslow low-dose t-PA. Thrombolysis with two cycles of the ultraslow high-dose t-PA regimen (100mg infused over 24 hours) ultimately resolved the thrombus burden, offering a viable non-surgical treatment.

Trials of higher doses of t-PA for mechanical PVT resistant to standard low-dose t-PA might be an unexplored therapeutic option before considering high-risk valve surgery. The PROMETEE trial found that ultraslow low-dose t-PA (25mg infused over 25 hours) was associated with low complications and mortality without compromising TT success [3]. The slower infusion allowed for earlier detection of complications and lowered the severity of complications when they occurred without decreasing thrombolytic efficacy. The TROIA trial evaluated the safety and efficacy of varying doses of t-PA, and showed good efficacy of high-dose t-PA (100mg) administered over six hours in treating PVT [13]. In our novel regimen, we administered high-dose (100mg) via an ultraslow infusion (24 hours) to combine the high efficacy of 100mg with the safety of slower infusions as demonstrated by the TROIA and PROMETEE trials [3,13].

Therefore, ultraslow infusion of the high-dose (100 mg) t-PA with a maximum dose of 200mg over 48 hours may balance high efficacy with a lower risk of adverse events and may be an acceptable alternative to surgery in cases of PVT that are refractory to standard management, especially in patients with preexisting hypercoagulable conditions. 

## Conclusions

Fibrinolysis with 100mg of t-PA infused over 24 hours may be considered for PVT unresponsive to repeat doses of guideline-recommended slow or ultraslow low-dose t-PA before proceeding to urgent surgical intervention. Though the risk of bleeding is higher with 100 mg t-PA than 25 mg, the ultraslow infusion may attenuate the risk. The decision of higher t-PA doses vs. urgent surgery requires a multidisciplinary approach and a shared decision-making process between the providers and the patient.
